# Expression of CK19 is an independent predictor of negative outcome for patients with squamous cell carcinoma of the tongue

**DOI:** 10.18632/oncotarget.12691

**Published:** 2016-10-15

**Authors:** Jutta Ernst, Kristian Ikenberg, Barbara Apel, Desiree M. Schumann, Gerhard Huber, Gabriela Studer, Tamara Rordorf, Oliver Riesterer, Matthias Rössle, Dimitri Korol, Marius G. Bredell

**Affiliations:** ^1^ Department of Cranio-, Maxillofacial and Oral Surgery, University Hospital Zurich, Zürich, Switzerland; ^2^ Department of Pathology, University Hospital Zurich, Zürich, Switzerland; ^3^ Department of Otorhinolaryngology, University Hospital Zurich, Zürich, Switzerland; ^4^ Department of Radiation Oncology, University Hospital Zurich, Zürich, Switzerland; ^5^ Department of Oncology, University Hospital Zurich, Zürich, Switzerland; ^6^ Department of Pathology, Cantonal Hospital Chur, Chur, Switzerland; ^7^ Cancer Registry Zurich and Zug, University Hospital Zurich, Zürich, Switzerland

**Keywords:** tongue cancer, cytokeratin 19 (CK19), Ki-67, head and neck cancer (HNC)

## Abstract

**Objectives:**

To explore the prognostic role of CK19 expression in squamous cell carcinomas within a well-defined cohort of oral tongue cancer patients.

**Methods:**

In our retrospective study, we investigated 129 patients with tongue cancer that had suitable material for inclusion in a tissue microarray (TMA). Where possible, samples were taken from central and peripheral regions of the tumor to generate a representative sample of the tumor. The expression level of CK19 was assessed by immunohistochemical staining.

**Results:**

Expression of CK19 in squamous cell carcinoma of the tongue was identified as a negative predictor for overall survival (OS; p<0.000) and disease specific survival (DSS; p=0.001). No significant difference could be shown for disease free survival (DFS) between patients with positive and negative CK19 staining (p=.094).

**Conclusion:**

This is the first description of the highly significant role of CK19 in a selective, organ specific head and neck cancer cohort. Our results are of special importance against the background that CK19 positive carcinomas revealed a significantly poorer prognosis and therefore emphasize its prognostic and possible diagnostic role in tongue cancer.

## INTRODUCTION

Head and neck squamous cell carcinoma (HNSCC) is the sixth most common type of malignancy worldwide [1] and also one of the most aggressive and invasive cancer types [2]. A common HNSCC hallmark is local invasion and metastasis to regional lymph nodes, accounting for up to 88% of patient mortality rate in the two years following metastatic disease development [3]. Despite the long-held notion of genomic instability in advanced disease stages, a recent study found no difference in the accumulation of mutations in tumors from patients with or without lymph node involvement [4]. This indicates that alterations other than mutations in signaling pathways likely account for the progression from primary tumor to invasive and metastatic disease. The contributions towards metastatic disease arise both from changes in the behavior of tumor cells and interactions with various stromal components in the tumor microenvironment [3].

In the past years, several efforts have been undertaken to define new biomarkers that will predict patient outcome and assist in patient-specific treatment adapted regimes [5, 6]. The identification of reliable tumor markers for HNSCC is expected to facilitate early diagnosis, predict prognosis as well as assist in therapy options and modifications of patients. Among this array of biomarkers Cytokeratin 19 and Cytokeratin-Fragment 19 (CK19, CYFRA 21-1), have emerged as two of the promising prognostic biomarkers [7, 8].

Cytokeratins (CK) are structural proteins forming the subunits of epithelial intermediary filaments. Twenty different CK polypeptides have thus far been identified. They are subgrouped into type I (40-56.5 kDa) and type II (53-67 kDa). Type I are acidic (CKs 9-20), type II are neutral to basic (CKs 1-8) [9]. Due to their specific distribution patterns, they are eminently suitable for use as differentiation markers in tumor pathology. CK19 protein is expressed in simple epithelia and their malignant counterparts. CYFRA 21-1 is released from malignant epithelial cells into human serum, tissue fluid, urine and saliva by a cleaving enzyme, caspase-3 during apoptosis [10] and can be detected with the aid of monoclonal antibodies. It has been shown to be a useful serum-based biomarker in lung and breast cancer [10, 11]. Moreover, CYFRA 21-1 is a prognostic-relevant marker for overall survival (OS) in metastatic colorectal cancer after selective internal radiation therapy [12].

Using CYFRA 21-1 to diagnose HNSCC has led to promising results. Wang et al. [13] showed in their meta-analysis that serum CYFRA 21-1 plays a role in the confirmation of HNSCC diagnosis, rather as a screening tool for HNSCC. Furthermore, they suggest that the level of CYFRA 21-1 indicates a positive correlation with the grade of differentiation and nodal status in HNSCC patients, and has a role in monitoring the success of therapy and follow up of patients [13]. In addition, an abrupt increase of CYFRA 21-1 in serial measurements during follow-up would likely indicate impending disease progression and provide early prognostic information, particularly with regards to tumor progression and metastatic formation in the individual HNSCC patient, regardless of the cut-off value. Therefore, the CYFRA 21-1 serum level is a good marker for follow-up in patients with HNSCC. In this way, CYFRA 21-1 may turn out to be useful not only for diagnosing HNSCC but also for characterizing its prognosis, which will improve the comprehensive management of HNSCC patients [13]. These suggestions are also supported by Zhong et al. [14], who found that CK19-positive score (staining intensity) in distant tissue (defined as at least 2 cm away from the edge of the cancerous mass) is predictive of tumor recurrence and survival in oral squamous cell carcinoma. Some patients with positive CK19 expression in distant tissue suffered from tumor recurrence and died of this disease during the follow-up period. Tumor recurrence rate is higher and survival rate lower in patients with positive CK19 expression in distant tissue than in those with negative CK19 expression in distant tissue. It can be suggested that positive CK19 expression in distant tissue may predict a higher risk of tumor recurrence and poor survival [14].

The aim of this paper is to explore the prognostic role of CK19 within a well-documented cohort of patients with oral tongue cancer, excluding tumors of the tongue base.

## RESULTS

The whole cohort consisted of 229 patients of which CK19 staining (positive versus negative) was available for 129 patients. Table 1 lists the patient demographics and tumor characteristics.

**Table 1:** Patient demographics and tumor characteristics. The Pearson's Chi-Square test revealed only a strong association between the expression of CK19 and alcohol consumption

	Total N	CK19 positive N (%)	CK19 negative N (%)	Pearson's Chi-Square
**Total**	129	41 (31.8)	88 (68.2)	
**Age**				p=0.883
<60	88	22 (53.7)	46 (52.3)	
≥60	41	19 (49.3)	42 (47.7)	
**Gender**				p=0.164
Male	80	29 (70.7)	51 (58)	
Female	49	12 (29.3)	37 (42)	
**Smoking Status**				p=0.062
Smokers	82	31 (75.6)	51 (58.6)	
Non-Smokers	46	10 (24.4)	36 (31.4)	
**Alcohol Consumption**				***p=0.018***
Risk Drinker BAG	29	12 (41.4)	17 (58.6)	
Non-Risk Drinker BAG	87	22 (25.3)	65 (74.7)	
Suspended or no information	11	7 (63.6)	4 (36.4)	
**Tumor Grading**				p=0.549
Well-differentiated	19	4 (9.8)	15 (17)	
Moderately differentiated	87	29 (70.7)	58 (65.9)	
Undifferentiated	23	8 (19.5)	15 (17.1)	
**Tumor Staging**				p=0.472
pT1	56	15 (41.7)	41 (48.8)	
pT2-4	65	21 (58.3)	44 (51.2)	
**Nodal Status**				p=0.153
pN0	71	16 (48.5)	48 (63.2)	
pN1-3	45	17 (51.5)	38 (36.8)	
**Local Recurrence**				p=0.224
Yes	16	3 (7.3)	13 (14.9)	
No	112	38 (92.7)	74 (85.1)	
**Nodal Recurrence**				p=0.098
Yes	14	7 (17.1)	7 (8)	
No	114	34 (82.9)	80 (92)	
**Perineural Invasion**				p=0.851
Yes	38	12 (34.3)	26 (32.5)	
No	77	23 (65.7)	54 (67.5)	

**Figure 1:**
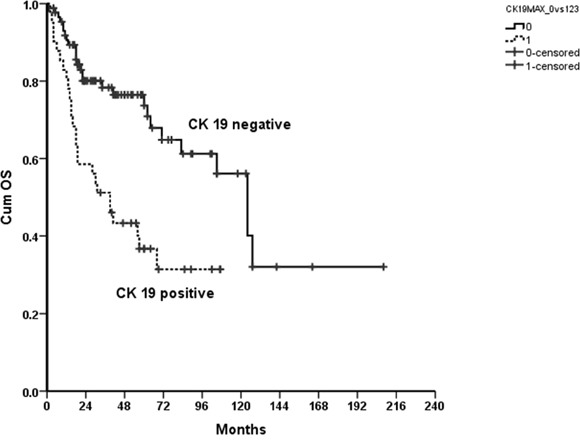
Kaplan Meier curve showing a statistically significantly improved OS for patients with negative compared to positive CK19 immunoreactivity of their tumors; p<0.00

We found that patients with positive CK19 immunoreactivity had a statistically significant worse outcome with regards to OS than patients with negative CK19 immunoreactivity (p<0.000; Figure 1). We investigated the survival differences in more detail and stratified for several demographic and prognostic factors as shown in Table 2. We found significant differences in gender (men, log rank, p =.002), age (≥60 years, log rank, p =.001), smoking (yes, log rank, p =.017; no, log-rank, p =.015) alcohol (risk drinker, log rank, p =.012), tumor staging (pT2-4b, log rank, p =.022), nodal status (pN1-3a, log rank, p =.027) tumor grading (2, log rank, p =.009) and perineural invasion (no, log rank, p <.000).

**Table 2:** Significant factors negatively affecting the outcome of OS

Risk	Men	≥60y	Smokers	Non Smokers	Risk Drinker	pT2-4b	pN1-3a	Tumor Grade 2	No Perineural Invasion
p	.002	.001	.017	.015	.012	.022	.027	.009	<.000

**Figure 2:**
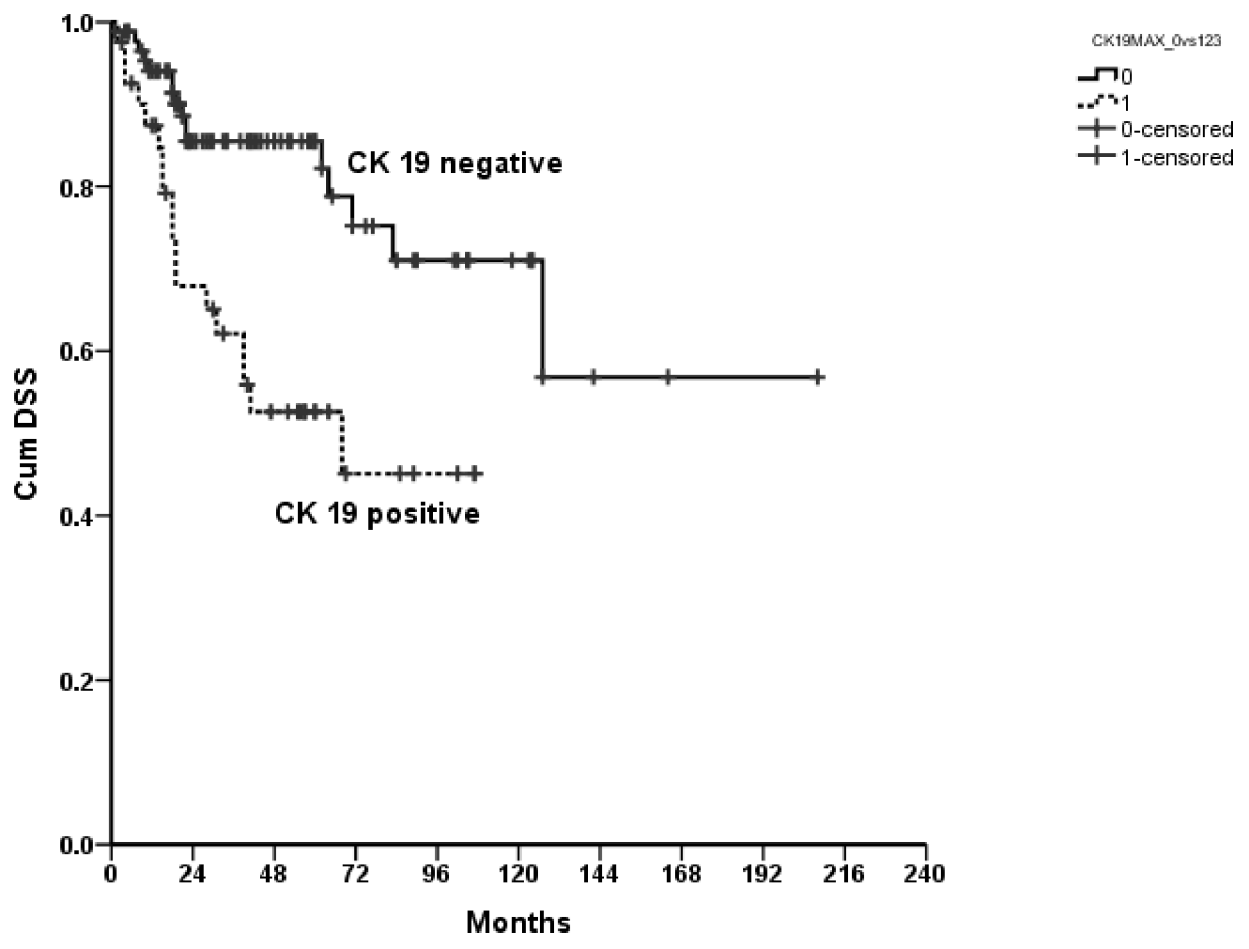
Kaplan Meier curve showing a statistically significantly improved disease specific survival for patients with negative compared to positive CK19 immunoreactivity of their tumors; p=0.001

Patients with positive CK19 immunoreactivity also had a significantly worse outcome regarding DSS than patients with negative CK19 immunoreactivity (p=0.001; Figure 2). In addition, DSS was affected by gender (men, log rank, p <.000), age (≥60 years, log rank, p =.001), smoking (yes, log rank, p =.040; no, log-rank, p =.010) alcohol (non risk drinker, log rank, p =.007), tumor staging (pT1, log rank, p =.011) and tumor grading (2, log rank, p =.036; Table 3).

**Table 3:** Significant negatively factors affecting the outcome of DSS

Risk	Men	≥60y	Smokers	Non Smokers	Non Risk Drinker	pT1	Tumor Grade 2
p	<.000	.001	.040	.010	.007	.011	.036

There is still a significant difference in OS between patients with positive and negative CK19 immunoreactivity, after adjustment for demographic (age, smoking and alcohol) and known prognostic factors (pT, pN, tumor grading) (p=.010, Exp(B)=2.0349, 95% CI 1.231-4.482).

A Cox-Regression showed that alcohol (p=.026, Exp(B)=1.666, 95% CI 1.098-3.069) and tumor grading (p=.024, Exp(B)=1.916, 95% CI 1.0601-3.049) were significant predictors for the differences in OS between patients with positive and patients with negative CK19 immunoreactivity, whereas in DSS no demographic or prognostic factor was a statistically significant risk factor.

No significant difference could be shown for DFS between patients with positive and negative CK19 immunoreactivity (p=.094).

To investigate a possible association between CK19 and other known predictive biomarkers in HNSCC we ran a Spearman's rho correlation and only found a significant correlation for CK19 and Ki-67 (p=.010, R=.227).

## DISCUSSION

CK19 is an acidic protein of 40 kDa that is part of the cytoskeleton of epithelial cells. The expression depends mainly on the epithelium type and on the status of epithelial terminal differentiation and maturation. Furthermore, this smallest known acidic cytokeratin is not paired with a basic cytokeratin in epithelial cells. It is specifically expressed in the periderm, the transiently superficial layer that envelops the developing epidermis [15]. CK19 is expressed in simple epithelium and the basal cells of squamous epithelium and is mainly interpreted as a marker of dysfunctional epithelial differentiation [8], however the underlying biology is still poorly understood.

The present retrospective study examined the effect of CK19 on overall and disease specific survival of an oral tongue cancer cohort. We found that patients with positive CK19 staining in oral tongue HNSCC had a worse outcome regarding overall and disease specific survival than patients with negative CK19 staining. Our findings are in line with other studies that found that increased CK19 expression in locally advanced HNSCC cases is related to decreased survival [14]. This is, however, the first organ specific study (solely oral tongue cancer patients) demonstrating the significant predictive value of CK19 in tumor tissue.

The mucosal lining of a large part of the human oral epithelium is non-keratinized, but can undergo abnormal keratinization in premalignant and malignant (e.g. squamous cell carcinoma) lesions of the oral mucosa. These changes in the patterns of keratinization are accompanied by changes in the expression of cytokeratins and other biomarkers. In oral epithelium, the hard palate, attached gingiva, and dorsal tongue are keratinized, but the oral floor and buccal region are non-keratinized. Lingual keratinized epithelial cells, which constitute the filiform papillae of the tongue, have one of the most rapid tissue turnover rates in the mammalian body and are thought to be the source cells of squamous cell carcinoma of the tongue [16].

Ki-67 is a well-known cellular marker for proliferation and is absent in the resting phase of cells. It was selected in this study as the most referenced biomarker for proliferation and we found a significant correlation between CK19 and Ki-67. This correlation could possibly shed light on tumor aggressiveness and therapeutic response since an increased proliferation rate (Ki-67) is indicative of a worse prognosis [18–20].

Human Papilloma Virus (HPV) is not regarded as a major pathogen in oral cancer and as only five patients in this cohort showed a positivity to p16 the relationship to CK19, HPV and smoking was not explored further [17]. Contrary to smoking, consuming alcohol appears to be associated with a higher expression of CK19. This has been seen in salivary glands of rats fed ethanol through gavage for 55 days [18]. A possible explanation may be the higher rate of dysplasia in alcohol users versus the Non Drinkers, however, further investigation is required [19].

CYFRA 21-1 is the soluble fragment of CK19 and is released once CK19 is cleaved by caspase-3 during apoptosis. Other studies showed a positive correlation between salivary and serum CYFRA 21-1 and CK19 mRNA and protein expression [9, 14]. In this study CK19 expression did not have a significant correlation to tumor size or tumor stage. This discrepancy between the positive correlation of CK 19 to poor OS, DSS and tumor proliferation rate (Ki-67) as well as non-correlation to tumor stage may indicate that CK 19 is a surrogate marker for invasiveness or tumor aggression. Moreover, our results are to a certain extent comparable to Malhotra et al., who found no correlation between CK19 mRNA levels and recurrence or tumor grade. However, our results do show a significant correlation to DSS which may implicate tumor persistence, recurrence or metastasis [9]. Though, the relationship between CK19 tissue expression and CYFRA 21-1 levels in body fluid is still heterogeneously discussed. CYFRA 21-1 levels in serum tend to be sensitive, but salivary CYFRA 21-1 appears to be the most sensitive, thus salivary CYFRA 21-1 levels combined with CK19 immunohistochemistry could reflect the prognosis more accurately [9].

In conclusion, our results from 129 patients with oral tongue cancer showed a highly significant association of CK19 positive immunoreactivity to overall survival and disease specific survival. We further found a significant correlation between CK19 and Ki-67 which indicates some interrelationship of CK19 to the higher turnover in certain tumors.

This is of special importance against the background that both high proliferation rate (Ki-67) and CK19 positive carcinomas revealed a significantly poorer prognosis that may indicate a more invasive and aggressive tumor behavior. The role of Ki-67 and CK19 and its serum fragments CYFRA 21-1 for use in individual tumor diagnosis and prognosis should be explored prospectively.

## MATERIALS AND METHODS

### Patients

A total of 229 tongue cancer (excluding tongue base) patients who fulfilled the inclusion criteria were identified in the data search. 129 patients had suitable material for inclusion in a tissue microarray (TMA) and were included in this retrospective study. Where possible, samples were taken from central and peripheral regions of the tumor to generate a representative sample of the tumor. All of the patients gave their consent for data search, use of biological material and further use of their data when they were admitted to the hospital. The study was approved by the Cantonal Ethic Commission Zurich (KEK-ZH-Nr. 2013-0298) and cause and dates of death were sourced from the Cancer Registry at the University Hospital Zurich. The median follow-up time was 36 months.

### Inclusion criteria

All tongue cancer patients, older than 18 years, treated at the Department of Cranio-, Maxillofacial and Oral Surgery and/or the Department of Otolaryngology, Head and Neck Surgery and/or the Department of Radiation-oncology and/or the Department of Medical Oncology at the University Hospital Zurich from 2005 to 2014 were included in the study. The defined oral tongue cancer did not include the base of the tongue and could be defined as primary cancer of the oral tongue and was distinguishable from primary floor of mouth cancer.

### Study variables

Clinical data and personal information pertaining to family history, and use of alcohol and tobacco were obtained through a comprehensive review of medical records. Alcohol consumption was divided into three groups: Drinkers, which included occasional as well as regular drinkers (Risk Drinker, Bundesamt für Gesundheit (BAG), Switzerland) and Non-Drinkers including patients who never drank alcohol (Non-Risk Drinker, BAG), or those who stopped drinking alcohol at least one year before diagnosis or no information was available. Smoking status was divided into two groups: Smokers, which included patients who smoked regardless of the pack years, and patients who stopped smoking less than one year before diagnosis and Non-Smokers including patients who had never smoked.

### Tissue microarrays and immunohistochemistry

Tissue microarrays were constructed from paraffin-embedded (FFPE) tissue as previously described [20]. Tumor tissue blocks were collected from the Institute of Surgical Pathology, University Hospital Zurich, Switzerland and selection was based on availability, quality and size of tumor tissue on the paraffin block.

**Figure 3:**
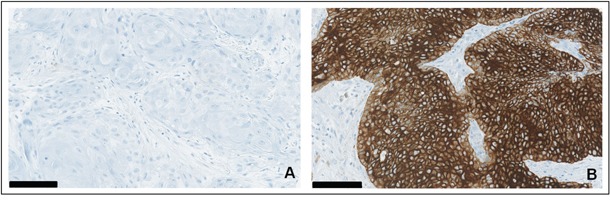
**A**–**B.** Expression of Cytokeratin 19 in oral squamous cell carcinoma: (A) immunohistochemical staining for CK19 with negative tumor tissue versus (B) strong positivity in other representative samples of malignant tumors, surrounding stromal tissue remains unstained. Scale bar = 100 μm.

For immunohistochemical studies, consecutive 3 μm-sections were freshly cut from paraffin TMA tissue blocks. Stainings were performed on an automated staining system (Ventana BenchMark ULTRA, Roche-Ventana Medical Systems, Tucson, AZ, USA) according to the manufacturers' instructions. Cytokeratin 19: Pretreatment Protease1 for 4min, mouse monoclonal anti-Cytokeratin 19 (RCK108) antibody (Abcam plc, Cambridge, UK) for 16min; Ki-67: Pretreatment Cell Conditioning 1 (CC1) for 32min, rabbit monoclonal anti-Ki-67 (30-9) antibody (Roche-Ventana Medical Systems, Tucson, AZ, USA). For analyses of Cytokeratin 19 a semi-quantitative four-step scoring system (0-3) was used based on the staining intensity of the tumor tissue: 0 = negative; 1 = weak positive; 2 = intermediate positive; 3 = strong positive. CK19 positive refers to scores 1-3 and CK19 negative to score 0 (Figure 3). For Ki-67 the percentage of stained nuclei of tumor cells was recorded.

### Statistical analysis

The primary endpoint was overall survival (OS) as measured as the time from the date of diagnosis to the date of death or date of last contact. Secondary endpoints were disease free survival (DFS) measured as the time from the end of treatment to date of relapse, recurrence or distant metastasis and disease specific survival (DSS), which was measured as the time from the date of diagnosis to the date of death where the cause of death was disease specific.

Pearson's Chi-Square was applied to evaluate how likely it is that any observed difference between the sets of categorical data arose by chance (p<.05). Survival was calculated using Kaplan-Meier curves. Multivariate analysis was performed using Cox Regression (p<.05). All statistical analyses were performed using SPSS 22 (IBM Corp. Released 2013. IBM SPSS Statistics for Windows, Version 22.0. Armonk, NY: IBM Corp.).
